# Supplementary material for: phastSim: Efficient simulation of sequence evolution for pandemic-scale datasets
Source: PLoS Comput Biol. 2022 Apr 29;18(4):e1010056. doi: 10.1371/journal.pcbi.1010056 (PMC9094560; doi:10.1371/journal.pcbi.1010056)
Supplement: S1 Text — Supplementary file containing results regarding memory demand of different methods and containing results from testing of phastSim features. (PDF) [file pcbi.1010056.s001.pdf]

Supporting Information File S1 for "phastSim: efficient simulation of sequence evolution for pandemic-scale datasets"

Nicola De Maio[1,*,☯], William Boulton[1,#a,☯], Lukas Weilguny[1], Conor R. Walker[1,2,#b], Yatish Turakhia[3], Russell Corbett-Detig[4,5], Nick Goldman[1],

1 European Molecular Biology Laboratory, European Bioinformatics Institute, Hinxton, UK
2 Department of Genetics, University of Cambridge, Cambridge, UK
3 Department of Electrical and Computer Engineering, University of California San Diego, San Diego, California, USA
4 Department of Biomolecular Engineering, University of California Santa Cruz, Santa Cruz, California, USA
5 Genomics Institute, University of California Santa Cruz, Santa Cruz, California, USA
#a Current Address: School of Computing Sciences, University of East Anglia, Norwich, UK
#b Current Address: New York Genome Center, New York, New York, USA

☯These authors contributed equally to this work.
* Contact: demaio@ebi.ac.uk

figureSS0 Fig.

Comparison of memory demand

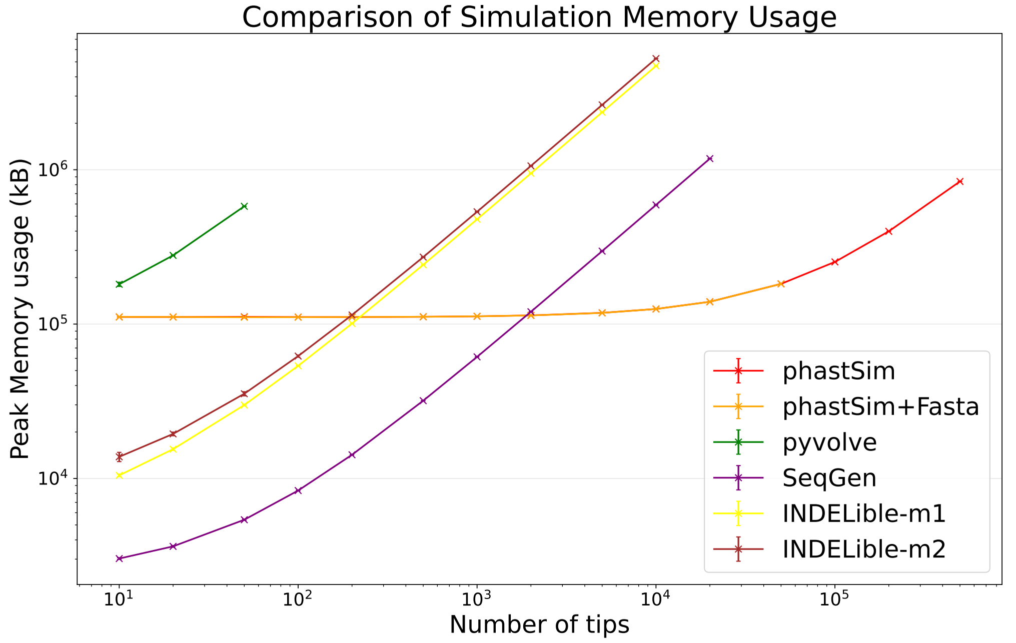

S1 Fig. Comparison of memory demand of different simulators in a scenario similar to SARS-CoV-2 data. On the Y axis we show the maximum memory demand in kB to perform simulations using different software. On the X axis is the number of tips simulated. Each point represents the mean of ten replicates. In red is the memory demand of phastSim with a concise output, and in orange of phastSim with additionally generating a FASTA format output (these values largely overlap). In green is the demand of pyvolve, and in purple of Seq-Gen. In yellow and brown are respectively the memory demand of INDELible with method 1 (matrix exponentiation) and method 2 (Gillespie approach).

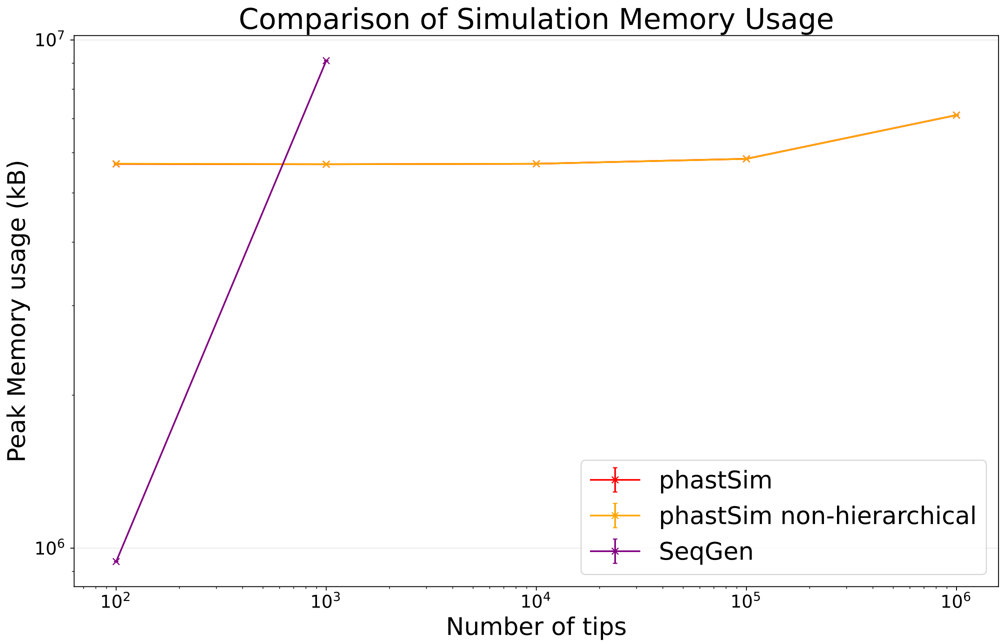

S2 Fig. Comparison of memory demand of different simulators in a scenario similar to *E. Coli* outbreak data. On the Y axis we show the memory demand in kB to perform simulations using different software. On the X axis is the number of tips simulated. Each point represents ten replicates. We do not run Seq-Gen for more than 1000 tips due to high computational demand. In red is the memory demand of phastSim, and in orange of phastSim with the simple non-hierarchical approach (values for the two largely overlap).In purple is the memory demand of Seq-Gen.

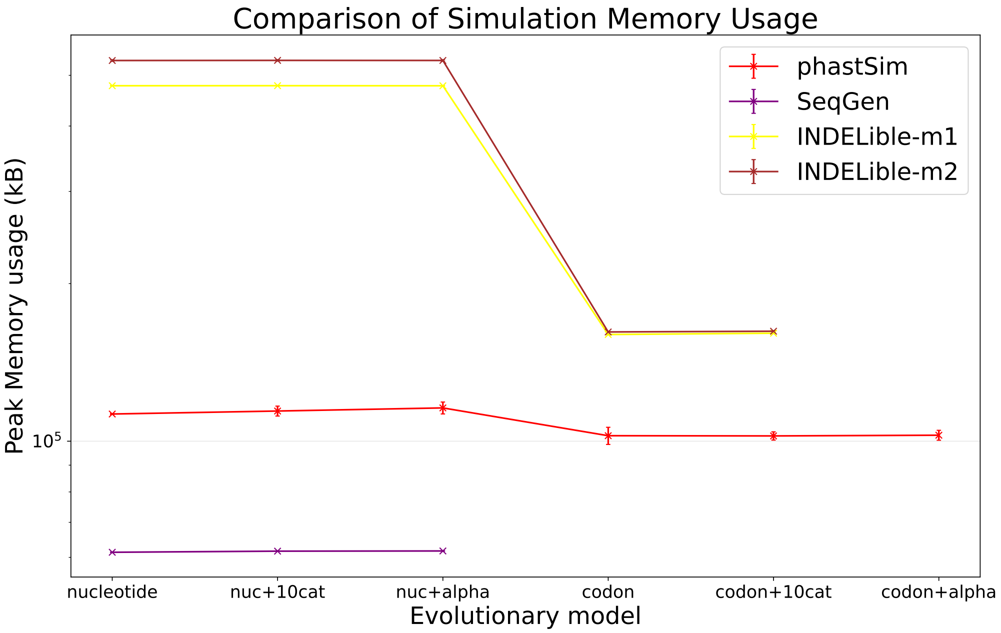

S3 Fig. Comparison of memory demand of different simulators in a SARS-CoV-2 scenario using different evolutionary models. On the Y axis we show the maximum memory demand in kB to perform simulations using different software. On the X axis is the model used for simulations: "nucleotide" is a nucleotide substitution model without variation; "nuc+10cat" is a nucleotide model with 10 rate categories; "nuc+alpha" is a nucleotide model with continuous variation in rate (each site has a distinct rate sampled from a Gamma distribution); "codon" represents a codon substitution model; "codon+10cat" represents a codon substitution model with 10 categories for ω; "codon+alpha" is a codon model with continuous rate variation in mutation rate and in ω (only allowed in phastSim). Each value represents ten replicates. Seq-Gen does not allow codon models. Here we used alignments of 1000 tips.

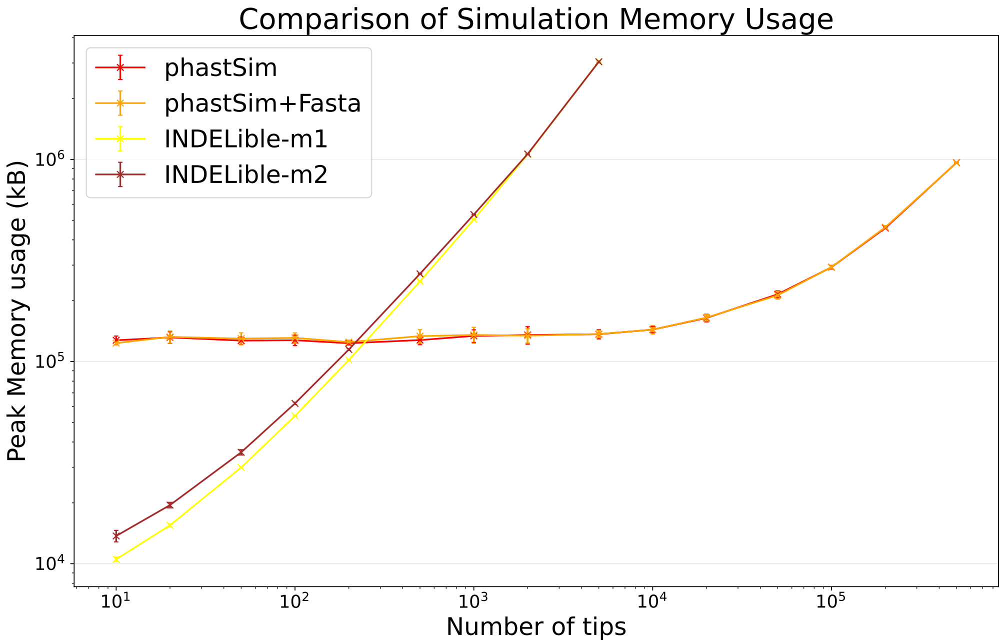

S4 Fig. Comparison of memory demand of Indelible and phastSim simulators in a SARS-CoV-2 scenario with indels. In this scenario we compare phastSim against Indelbile-m1 and Indelible-m2 (the only other methods considered here that model indels). Each point represents ten replicates.

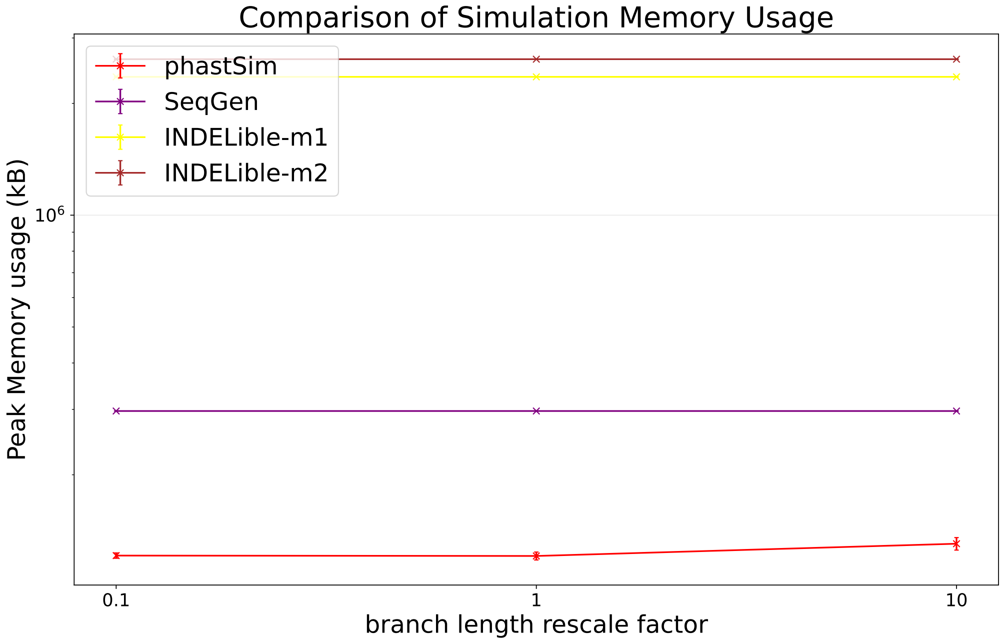

S5 Fig. Comparison of memory demand of different simulators in a SARS-CoV-2 scenario after rescaling the tree branch lengths by different factors. On the Y axis we show the maximum memory demand in kB to perform simulations using different software. On the X axis is the rescaling factor we use to make the phylogenetic tree branch lengths longer or shorter. Here we used alignments of 5000 tips.

Testing the correctness of phastSim simulations

Tree-likeness of simulated alignments and correctness of the simulated substitution process

We ran a set of systematic simulations to assess if the genomes simulated by phastSim adhered to the evolutionary history represented by the input phylogenetic tree, and if the substitution process simulated by phastSim well represents the one specified by the user. To do this, we simulated 100 replicates each with a random trees with 8 tips and random branch lengths between 0.0005 and 0.0015, simulated with ETE3 [1]. For each replicate we simulated genome evolution with phastSim under a GTR model with random substitution rates (uniformly sampled between 0 and 1) but constant nucleotide frequencies (0.1, 0.2, 0.3, and 0.4 respectively for A, C, G and T); the substitution matrix was normalized as usually done in phylogenetics before simulating sequence evolution. We did not simulate indels. A random root genome sequence of length 10^6 bases was sampled for each replicate according to the equilibrium nucleotide frequencies.

Then, for each of the 100 replicates, we ran RAxML v8.2.11 (raxmlHPC) [2] with a GTR model and no rate variation. The trees inferred by RaxML were always identical in topology to the ones simulated. Furthermore, the total estimated length of the tree closely matched the simulated one (S6A Fig, difference below 2.0% of the simulated value) as did the substitution rates (S7A Fig, errors between 0.5 and 3%) and the equilibrium nucleotide frequencies (S7B Fig, errors between 0.7 and 0.8%).

Correctness of the simulated substitution rate variation

Here we wanted to test the correctness of the simulated rate variation across sites in phastSim. We simulated 100 trees, each relating 128 samples, as in the previous section, but with branch lengths uniformly sampled from the interval $[0.05, 0.15]$. For each tree, we then simulated an alignment using phastSim under a JC69 model and with root genome 1000 bp long. We simulated 25% of the sites as invariable. We then ran phylogenetic estimation with RAxML with a JC model and only inferred the tree and the proportion of invariant sites. The 100 inferred values for the proportion of invariable sites are shown in S6B Fig (inferred values between 22% and 29%).

In a second set of simulations of rate variation we used the same setting as above but simulated continuous rate variation across sites in phastSim under a gamma model with $\alpha = 1.5$. We then ran inference of tree and α with RAxML-NG v1.0.2 [3] under a JC69 model with a gamma model of rate variation with 20 discretized categories. The 100 values of α inferred by RAxML-NG are given in S6C Fig (inferred values between 1.3 and 1.7).

In the third set of simulations of rate variation we used again the same setting as above but simulated rate variation according to 3 site rate categories with frequencies of 0.25, 0.5 and 0.25, and rates of 0.1, 1.0 and 10.0, using phastSim. We then ran inference of tree and of the 3 site category frequencies and rates with RAxML-NG under a JC69 model. The squared errors of the 100 vectors of values of the categories rates and frequencies inferred by RAxML-NG are given in S7C Fig and S7D Fig.

Correctness of the simulated indel process

To test the correctness of the distribution of simulated indels, we simulate indels in phastSim and compare their distribution with those simulated by INDELible [4] (method 1) using the same indel distribution parameters. In the base simulation scenario, indels where simulated under a nucleotide model and along a tree with one ancestral sample and one descendant sample, with the two samples separated by a

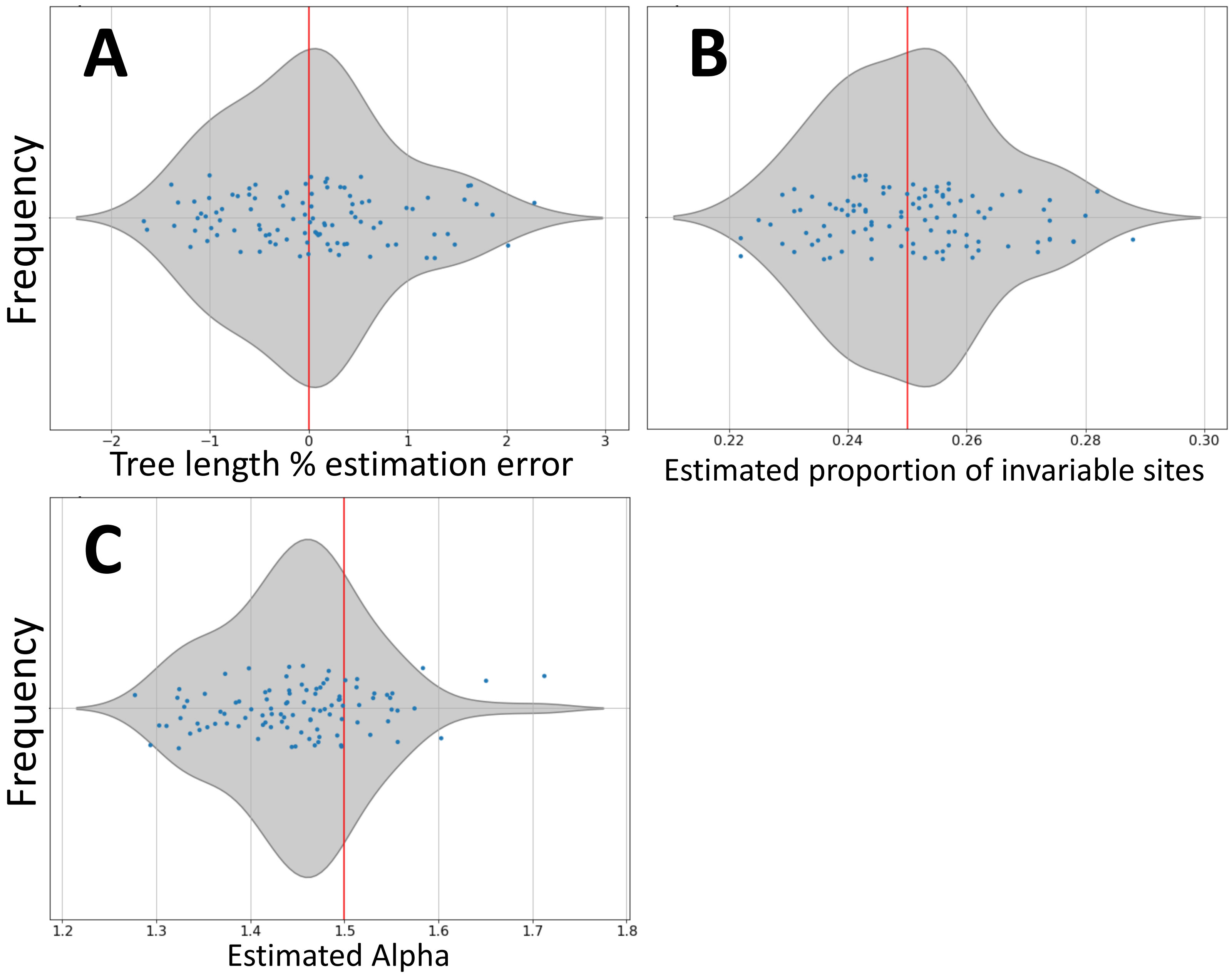

S6 Fig. RAxML and RAxML-NG estimations from phastSim simulations. Each point represents one of 100 RAxML (for **A** and **B**) or RAxML-NG (for **C**) estimations, each from a distinct dataset simulated by phastSim. Red vertical bars represent perfect estimates, corresponding to simulated values. **A** Difference between estimated and simulated tree length (the sum of all the branch lengths in the tree), expressed as a percentage of the simulated value. **B** Inferred proportion of invariant sites (the simulated value was 25%). **C** Estimated α parameter representing variation in substitution rates across the genome (the simulated value was $\alpha = 1.5$); part of the discordance between simulated and estimated value in **C** is likely due to the fact that we simulated continuous variation in substitution rates, which was approximated by RAxML-NG with 20 discretized rate categories.

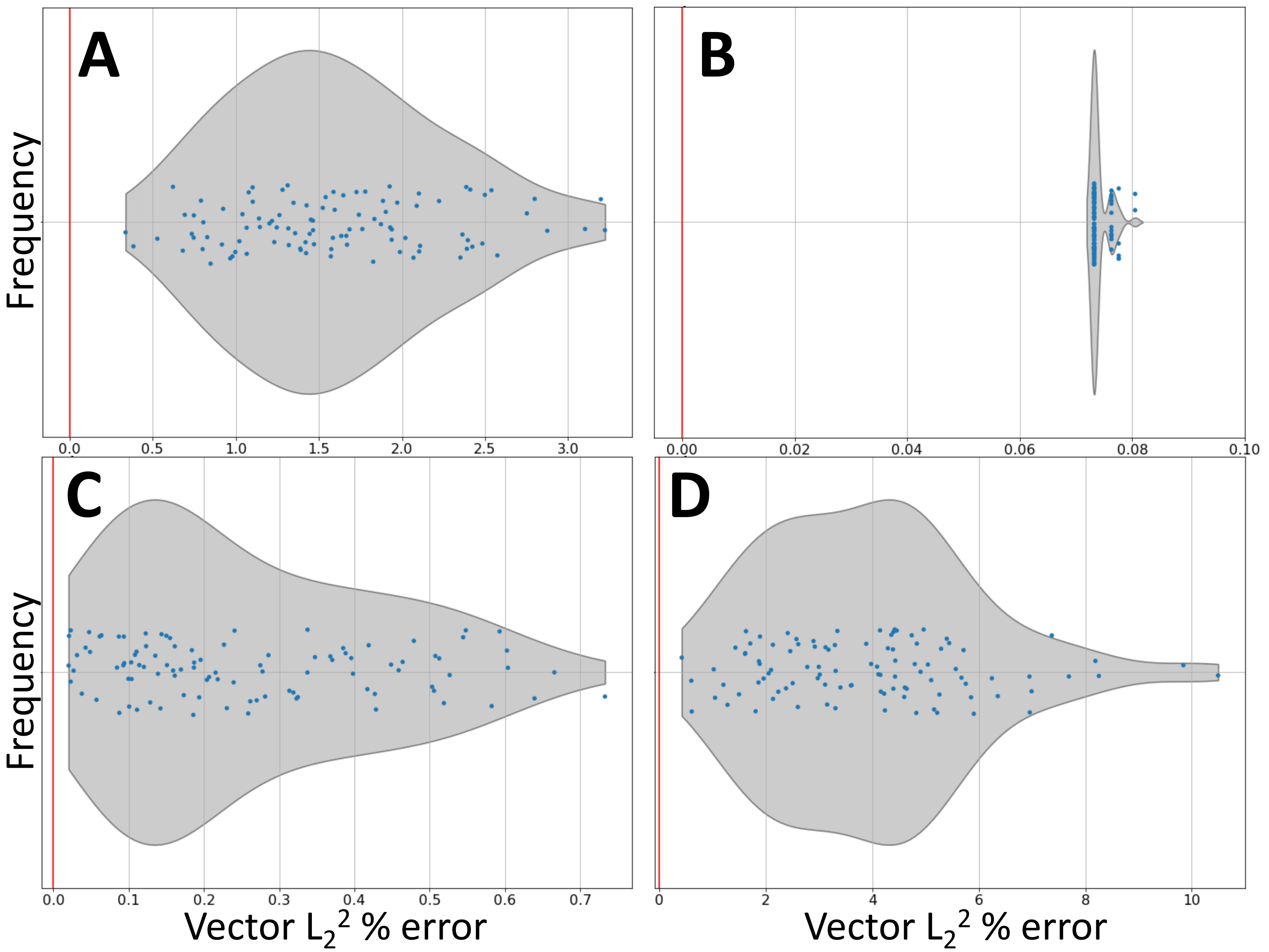

S7 Fig. RAxML and RAxML-NG estimation errors from phastSim simulations. Each point represents estimation errors from one of 100 RAxML (for **A** and **B**) or RAxML-NG (for **C** and **D**) estimations, each from a distinct dataset simulated by phastSim. Red vertical bars at 0 represent the absence of estimation error. Error is expressed as the square of the euclidean distance between simulated and estimated parameter vectors, and is scaled as percentage points. **A** Estimation of GTR substitution rates. The substitution rate parameter vectors were normalized so that the sum of the values within each vector was 1.0 before being compared. **B** Estimation of equilibrium nucleotide frequencies; estimates are very accurate, and most of the error shown is due to decimal number representation accuracy in RAxML. **C** Estimated substitution category rates - we simulated alignments with 3 site rate categories, with rates $(0.1, 1.0, 10.0)$ and frequencies $(0.25, 0.5, 0.25)$. **D** Estimated proportions of substitution categories, simulated as in **C**.

branch of length 10^{-4} substitutions per site (newick tree format "(S1:0.0001,S2:0.0);"). In the base scenario, insertion and deletion rates were both equal to the substitution rate, and indel lengths were geometrically distributed with parameter 0.3 (mean length 10/3). In addition to the base scenario, we consider 5 further modified scenarios:

- "Longer branch", same as the base scenario but with a 3 times longer branch separating the two samples.
- "More insertions", same as the base scenario but with 3 times higher insertion rate.
- "More deletions", with 3 times higher deletion rate.
- "Longer insertions", same as the base scenario but with 3 times longer (on average) insertions.
- "Longer deletions", with 3 times longer (on average) deletions.

For each scenario we ran 50 replicates for each of the two software, always using a root genome of 10^6 nucleotides. In addition to graphically comparing the simulated mean indel lengths and numbers across replicates (S8 Fig), we also ran 24 Mann-Whitney U Tests, one for each of the 6 simulation scenarios and each of the 4 statistics considered for each replicate (number of insertions, number of deletions, mean length of insertions, mean length of deletions). Of the 24 tests, and without applying multiple testing correction, only one comparison had a p-value below 0.05, the comparison of mean insertion lengths in the scenario of longer insertions (p-value 0.040566).

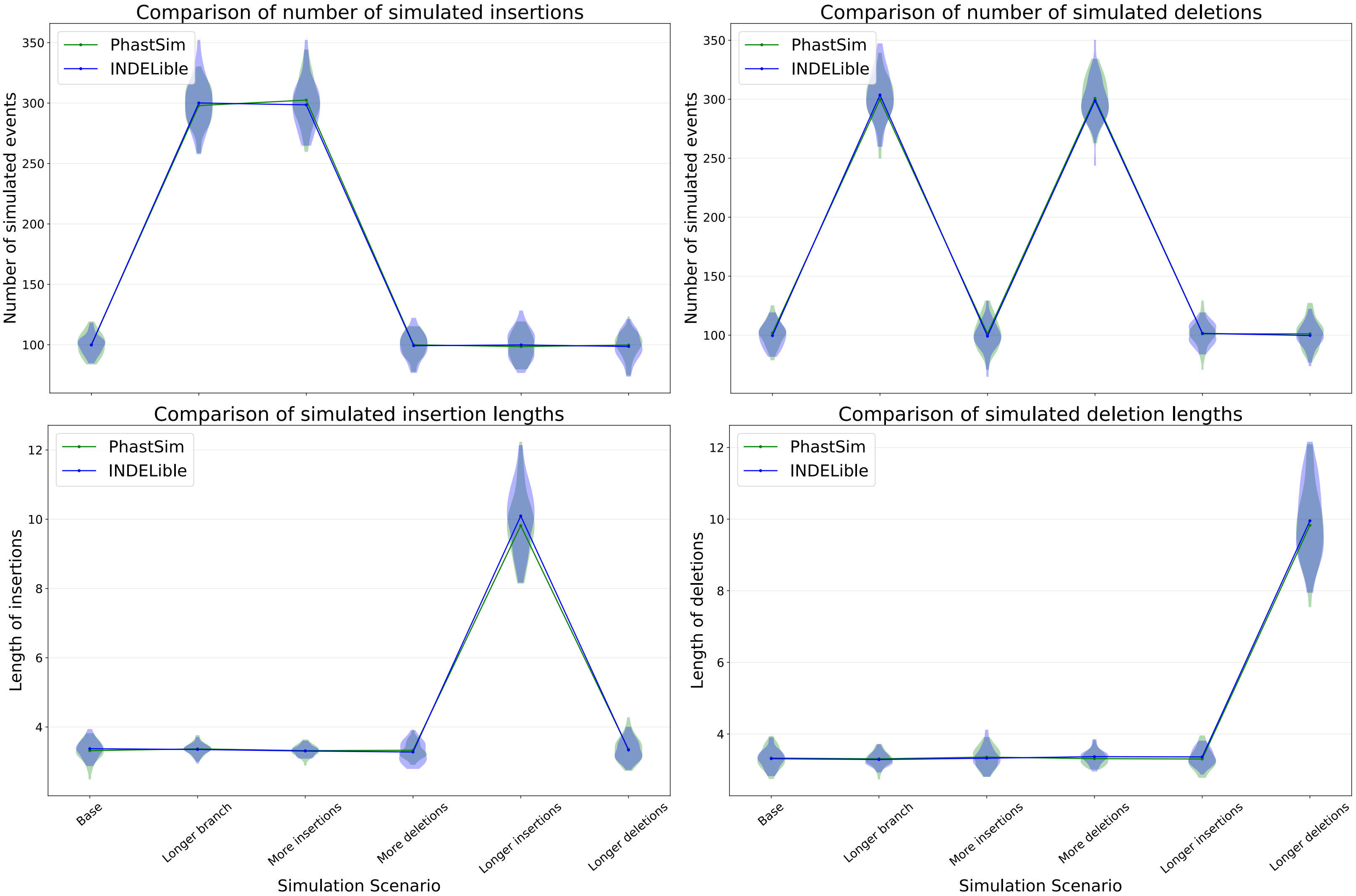

S8 Fig. Testing the correctness of simulated indel distributions. Here we simulated sequences using INDELible and phastSim in the scenarios described in the 6 indel scenarios described in the text. Each violinplot represents a distribution of 50 values corresponding to 50 simulation replicates. Dots (connected by lines) represent the mean of the distributions. Top plots represent the numbers of simulated indels in each replicate, while the bottom plots represent the average lengths of indels for each replicate. Values of the two methods mostly overlap.

References

1. Huerta-Cepas J, Serra F, Bork P. ETE 3: reconstruction, analysis, and visualization of phylogenomic data. Molecular biology and evolution. 2016;33(6):1635–1638.

2. Stamatakis A. RAxML version 8: a tool for phylogenetic analysis and post-analysis of large phylogenies. Bioinformatics. 2014;30(9):1312–1313.

3. Kozlov A, Darriba D, Flouri T, Morel B, Stamatakis A. RAxML-NG: a fast, scalable and user-friendly tool for maximum likelihood phylogenetic inference. Bioinformatics. 2019;35(21):4453–4455

4. Fletcher W, Yang Z. INDELible: a flexible simulator of biological sequence evolution. Molecular biology and evolution. 2009;26(8):1879–1888.
