# Supplementary material for: phastSim: Efficient simulation of sequence evolution for pandemic-scale datasets
Source: PLoS Comput Biol. 2022 Apr 29;18(4):e1010056. doi: 10.1371/journal.pcbi.1010056 (PMC9094560; doi:10.1371/journal.pcbi.1010056)
Supplement: Attachment — Submitted filename: phastSim review.pdf [file pcbi.1010056.s002.pdf]

The authors present **phastSim**, a new sequence simulation platform for simulating large datasets realistic to SARS-CoV-2 evolution, including both algorithmic advances and new simulation parameters (eg hypermutability). Overall I find this manuscript timely and well-written, with only a few minor comments -

- I really do not think the use of the quoted term "vanilla" method is appropriate. Quotes like this imply a lack of precision in defining what exactly "vanilla" means, and precision is very important in reporting scientific results. Further ,as far as I know from a bit of googling, "vanilla" was introduced to be used in the English language in this manner (i.e. not a bean/flavor) to indicate, well, so-called boring sexual practices. This is not the connotation one wants in a scientific manuscript. I encourage the authors to nail down what PRECISELY they mean by "vanilla" and use corresponding precise terminology throughout.
- I may have missed this in the manuscript, but what exactly is the formal relationship between the given branch lengths and how authors are considering the overall substitution Rd + Ri + Rs? In most cases, branch lengths will represent substitutions, but the model here proposes that changes are proposed one-at-a-time as either indel or substitution until the branch length is used up via the Gillespie approach. Are indel changes therefore considered part of the overall branch length?
- A further question about indels: What is the model that insertions follow after they've been inserted? A description about how the model applied for inserted sequences is parameterized will be helpful.
- Table 2: This is not correct for the pyvolve software. For codon models, pyvolve also contains MG94-style models (allowing for nucleotide frequencies instead of codon frequencies) as well as mutation-selection style codon models. Notably, pyvolve also includes an extension of mutation-selection models at the nucleotide level.
- It seems like the presented extended GY94 is actually much more similar to MG94. The main difference between these two models is not just including a separate dS parameter, but also the treatment of target frequencies. The matrix on page 14 suggests target nucleotide frequencies (as embedded in the applied mutation matrix) are being used, which is the MG94 model with dS fixed to 1.
- Regarding the benchmarks with other softwares, it's not surprising at all that pyvolve is the slowest of the bunch (as the author of pyvolve, I'm pretty comfortable with this - it was very much not written with efficiency in mind at all…). But, I will note that pyvolve also implements Gillespie and this may affect just *how* slowly it runs, though it is sure to be rather slow! It would be helpful to specify whether the benchmark used Gillespie or not. I see this script in the linked github - https://github.com/NicolaDM/phastSim/blob/main/scripts/runPyvolve.py - which does not specify Gillespie. If one wanted to, can add argument `algorithm = 1` when calling the evolver instance, e.g. `my_evolver(seqfile=pathSimu+outputFile, algorithm = 1)` in

version >= 1.0.0. Perhaps it could make your manuscript a bit stronger by showing, "Even with two different modes of simulating with pyvolve, it's still unreasonably slow!" :)
