# Supplementary material for: phastSim: Efficient simulation of sequence evolution for pandemic-scale datasets
Source: PLoS Comput Biol. 2022 Apr 29;18(4):e1010056. doi: 10.1371/journal.pcbi.1010056 (PMC9094560; doi:10.1371/journal.pcbi.1010056)
Supplement: Attachment — Submitted filename: phastSim_ResponseToReviewers.pdf [file pcbi.1010056.s003.pdf]

phastSim: efficient simulation of sequence evolution forpandemic-scale datasets

Response to the Decision Letter

Please find below in blue font our response to the comments from the Reviewers and the Editor.

Associate Editor: Joel O. Wertheim

Comments to the Author:

Thank you very much for submitting your manuscript ”phastSim: efficient simulation of sequence evolution for pandemic-scale datasets” for consideration at PLOS Computational Biology.

As with all papers reviewed by the journal, your manuscript was reviewed by members of the editorial board and by several independent reviewers. In light of the reviews (below this email), we would like to invite the resubmission of a significantly-revised version that takes into account the reviewers’ comments.

I agree with the consensus among the three reviewers. This approach is novel and potentially quite useful. That said, the reviewers identified several areas that require attention and improvement. In particular, Reviewers 1 and 3 raise an important point about the structure and focus of the manuscript.

Answer: We thank the Editor for the feedback. We have now addressed the concerns of the Reviewers. In particular, the new version of the manuscript has a new structure and additional simulations, as suggested by the Reviewers and Editor.

Reviewers’ Comments:

Referee: 1

In this manuscript, the authors present a novel tool for simulating the evolution of an ancestral sequence along a given phylogeny. The authors have made the tool

available as an open source package available on GitHub with user-friendly installation possible via PyPI (using the pip package manager).

As promised in the manuscript and GitHub repo, I was able to easily install the tool, along with all of its dependencies, on my laptop via a simple ”pip install phastSim” command (using an Ubuntu 18.04 environment in the Windows Subsystem for Linux). I was then able to run the tool using the example dataset provided in the GitHub repo, which finished running in just 15 seconds on my laptop, which is quite impressive given the sample dataset size. I only briefly skimmed through the code base on GitHub, but at a glance, it's quite clean and organized. Regarding the algorithms presented, the authors present clever techniques for efficiently simulating sequence evolution along a tree, and importantly, their approach supports the simulation of insertions and deletions, something not supported by Pyvolve nor (if I recall correctly) Seq-Gen.

Answer: We are very grateful to the Reviewer for the comments and suggestions; these are addressed below. Indeed, Seq-Gen does not support indels.

Regarding the manuscript itself, I believe the paper is overall well-written: as expected from a paper of this nature, the authors (1) introduce the bioinformatics problem at hand, (2) discuss prior work in the space, (3) introduce their novel approach, (4) present their approach's algorithms and tool implementation, (5) describe a simulation experiment to benchmark their tool against existing methods, (6) present the results of the benchmarking experiment, and (7) discuss the results. However, I believe the paper requires some significant revision:

- The technical details of the simulation experiment are currently presented in the ”Results” section of the manuscript. From my perspective, it would make more sense to move the technical details about the methods behind the simulation experiment to the ”Materials and methods” section of the manuscript. I believe only the results of the simulation experiment (i.e., the actual benchmarking measurements) should be presented in the ”Results” section

Answer: We thank the Reviewer for the suggestion, we have now reorganized the manuscript so that the datasets and software used for the comparison are described in the “Material and Methods” section and not in the “Results” one.

- The paper only shows runtime measurements for the various tools, but because of the large number of simulations that need to be executed, ideally with many replicates in parallel, in large-scale simulation experiments such as those used to study COVID-19 (e.g. Pekar et al., Science 2021), and because the simulation of sequence evolution can be quite memory-intensive (as mentioned by the authors), the benchmarking results should include plots depicting peak memory usage of the various tools as well. I apologize in advance for asking for this, as I'm sure it'll require quite of work to be redone, but in addition to runtime measurements, peak memory measurements are critical to properly compare these tools

Answer: We agree and think this is a good suggestion. We have now re-done the simulations keeping track of memory usage as well as run time, and included the new results in the manuscript (Figures 3-7) and supplementary material (S1 Text, Figures S1-S5). PhastSim relatively performance in terms of memory appears even better than the time one.

- Figure 1 should be cleaned up to look a bit more professional / production-quality.

For example, the child branches coming out of the internal nodes of the tree are quite inconsistent in terms of spacing, and rather than using "->" to denote a right arrow, it would be better to actually use a right arrow (→), etc.

Answer: We thank the reviewer for their suggestions about the figures of the manuscript. In our revision we include a substantially updated version of Figure 1 to address this issue.

- Figure 2 should be cleaned up substantially: the image looks quite distorted (namely the small red-and-blue trees), perhaps because of resizing vertically?

Answer: Our revised manuscript includes an updated version of Figure 2. Overall, we improved the consistency, and more specifically we changed the way the projections of the genome tree to its respective layers are presented. These projections avoid the trees appearing as vertically resized and should improve the clarity of the underlying methods.

- Figures 4, 5, and 7 should be redone to look consistent with Figures 3 and 6 (especially the legends and tick labels). Further, these 3 figures have far too much vertical space: the y-max should be much smaller (e.g. 150 seconds for Figure 4, 3 seconds for Figure 5, and 45 seconds for Figure 7)

Answer: We have now fixed these issues in the new versions of Figures 4, 5 and 7.

- Why are Figures 4, 5, and 7 using different-sized trees for each tool in these experiments? These should be replaced with the exact same trees for each tool, just as was done in Figures 3 and 6. If the decision to use different-sized trees was for the sake of presentation due to huge variation in runtime across the tools, the authors can use a log-scale for the vertical axis. Even in the figures' current form, the boxes are quite squished, and log-scale may help better depict them

Answer: We have now re-run the simulations (to track also memory demand) and have re-plotted the figures using the same number of tips for the different methods and using a log Y axis.

- Figure 5's minimum vertical axis value (y-min) should be 0, not -1, as these are runtimes (if the vertical axis is changed to log-scale as I recommended in a prior bullet, the y-min would need to be a positive number rather than 0)

Answer: We now use a logarithmic scale.

- I was not able to find the datasets used in the simulation experiments. I understand that GISAID has tight restrictions on releasing actual sequences, but the phylogenies used in the simulation experiments, along with the raw benchmarking measurements should be made publicly available (e.g. in a separate GitHub repo, on Data Dryad, on figshare, etc.). If the authors are worried about GISAID terms with respect to the phylogeny, the only identifiable component would be the tip labels, so the authors can simply replace the tip labels with arbitrary values (e.g. "0", "1", etc.). I would recommend also including all scripts/commands utilized in conducting the benchmarking experiments so that a reader can simply copy-and-paste the exact

commands you used and (more-or-less) reproduce the benchmarking results

Answer: Because of the reasons mentioned by the Reviewer, we did not use datasets from GISAID, but we instead simulated the phylogenetic trees, which then we used as input for phastSim and other simulators, using a custom script. To do this, we modified an existing tree simulation software (NGESH) to increase its efficiency; this modification is now included as an option in the software distribution (`https://github.com/tresoldi/ngesh`). All scripts used for simulations and plotting are included in the phastSim GitHub repository `https://github.com/NicolaDM/phastSim/tree/main/scripts`. We also include the bash scripts that we used to run the comparisons on the cluster, so the whole experiment could be repeated by using these bash scripts after appropriate installations.

Less significant general comments for improvement of presentation:

- The formatting of the pseudocode in the various algorithms is somewhat inconsistent. Of note, the spacing between the equal signs in assignments is inconsistent (sometimes "a=b", sometimes "a= b", sometimes "a =b", and sometimes "a = b"). It would be good to revise to be consistent; I would recommend putting spaces between symbols for clarity

Answer: We have now increased the spacing overall and made it consistent across all the pseudocode as suggested by the Reviewer.

- Assignment operations in pseudocode are typically denoted using a left arrow (←) rather than using an equal sign (=)

Answer: We have now modified this as suggested.

- Multiple parts of the paper say "sample ___ from an exponential distribution with parameter ____", which is slightly ambiguous: the exponential distribution has two possible parameterizations (rate, or scale = 1/rate), and while the rate parameterization is the most typical representation (to my knowledge), it would be good to specify, e.g. "sample ____ from an exponential distribution with rate parameter ____"

Answer: We have now clarified this.

- All figures appear quite pixelated in the PDF I downloaded. However, this may be an artifact of the submission system, so it may not actually be an issue on the authors' end (but it would be good to double check)

Answer: Our original figures are in pdf format and not pixelated - so there must have been an issue when converting our pdf figures into formats accepted by the submission system. We will try to solve the issue this time.

- Figure 3 may be improved by presenting the vertical axis in log-scale to better distinguish between the runtimes of smaller values of "number of tips" (though not necessary, as the trends are quite clear even in the current presentation)

Answer: We now use logarithmic y axes on all the figures.

Specific comments about wording/grammar/text throughout the manuscript:

- ”Sequence simulators are fundamental tools in bioinformatics, as they allow us to test data processing and inference tools, as well as being part of some inference methods” → The last clause of this sentence is grammatically incorrect and should be revised
- ”Here we present a new algorithm and software for ...” → There should be a comma after ”Here”
- ”Our algorithm is based on the Gillespie approach, and implements an ...” → There should be an ”it” before ”implements”
- ”either for example through Approximate Bayesian Computation [6, 7], see e.g. [8, 9],” → I wonder if this could just be changed to be ”Approximate Bayesian Computation [6-9]” (i.e., remove the ”e.g.” part)? Same comment for the following sentence
- The paragraph starting with ”In this simplified “vanilla” scenario...” may benefit from being split into two parts, e.g. with a new paragraph starting at ” A pseudocode description of...”
- The end of the paragraph starting with ”In this simplified “vanilla” scenario...” presents details about the tool implementation, though those tool-specific descriptions should likely be moved to the portion of the manuscript that describes the tool
- There are some more minor grammar issues throughout, so the paper may benefit from another pass of internal revisions for such things

Answer: We have now included these corrections and we have tried to fix grammatical errors.

Overall, I was thoroughly impressed by this work, and I look forward to utilizing phastSim in my own research!

Answer: We are very grateful to the Reviewer for the comments and suggestions, which we hope to have addressed in this new version of the manuscript.

Referee: 2

Comments to the authors

The authors present phastSim, a new sequence simulation platform for simulating large datasets realistic to SARS-CoV-2 evolution, including both algorithmic advances and new simulation parameters (eg hypermutability). Overall I find this manuscript timely and well-written, with only a few minor comments -

Answer: We thank the Reviewer for the comments, which we address below.

I really do not think the use of the quoted term ”vanilla” method is appropriate. Quotes like this imply a lack of precision in defining what exactly ”vanilla” means, and

precision is very important in reporting scientific results. Further ,as far as I know from a bit of googling, ”vanilla” was introduced to be used in the English language in this manner (i.e. not a bean/flavor) to indicate, well, so-called boring sexual practices. This is not the connotation one wants in a scientific manuscript. I encourage the authors to nail down what PRECISELY they mean by ”vanilla” and use corresponding precise terminology throughout.

Answer: We understand the point, so we have now modified the manuscript to get rid of the term “vanilla”, and replaced it with “simple” and “non-hierarchical” throughout.

I may have missed this in the manuscript, but what exactly is the formal relationship between the given branch lengths and how authors are considering the overall substitution Rd + Ri + Rs? In most cases, branch lengths will represent substitutions, but the model here proposes that changes are proposed one-at-a-time as either indel or substitution until the branch length is used up via the Gillespie approach. Are indel changes therefore considered part of the overall branch length?

Answer: We measure branch lengths in terms of expected number of substitutions per site for the root genome. This means that indel rates are not considered when defining branch lengths. It also means that the expected number of substitutions for a branch length of 1 might not be one far from the root, and, in fact, typically the expected number of substitutions per unit of branch length will decrease as one moves away from the root, in particular with models with extreme variation in mutation rates. The reason for this is that we do not necessarily assume that the root genome nucleotide frequencies are necessarily the equilibrium frequencies for the considered substitution rates. We think that our approach for defining branch lengths makes sense, since otherwise one would have that at each mutation event the meaning of branch lengths would change, which we think would make things more confusing and hard to interpret. An alternative could be to define branch lengths using expected mutations at equilibrium, but expected mutations at equilibrium might differ significantly from those in the phylogeny, since SARS-CoV-2 composition appears substantially distant from equilibrium considering the viral substitution process observed within humans (see e.g. `https://doi.org/10.1093/gbe/evab087`). We have now rephrased the content of Section “Rate normalization” to make these aspects more clear.

A further question about indels: What is the model that insertions follow after they’ve been inserted? A description about how the model applied for inserted sequences is parameterized will be helpful.

Answer: Indeed we did not mention this aspect of the model in the earlier version of the manuscript. If the user specifies a root genome, inserted nucleotides are randomly and independently sampled from the root genome nucleotide frequencies; if the user does not specifies a root genome, but instead specifies root nucleotide frequencies for phastSim to sample a random root genome from them, then the same sampling is done for inserted sequences. The substitution model for each inserted nucleotide/codon is chosen at the time of insertion in the same way as for the root genome (in particular also accounting for rate variation). We now include this information in the section “Insertion Algorithm”.

Table 2: This is not correct for the pyvolve software. For codon models, pyvolve also

contains MG94-style models (allowing for nucleotide frequencies instead of codon frequencies) as well as mutation-selection style codon models. Notably, pyvolve also includes an extension of mutation-selection models at the nucleotide level.

Answer: Indeed we underrepresented the choice of codon model for pyvolve. We have now rephrased this to "GY94, MutSel and MG94-style" in Table 2.

It seems like the presented extended GY94 is actually much more similar to MG94. The main difference between these two models is not just including a separate dS parameter, but also the treatment of target frequencies. The matrix on page 14 suggests target nucleotide frequencies (as embedded in the applied mutation matrix) are being used, which is the MG94 model with dS fixed to 1.

Answer: It is true that the our model implies equilibrium nucleotide frequencies, and that these have a similar interpretation as in the MG94; however, our mutation rates are not only defined by the nucleotide equilibrium frequencies, but also by the underlying UNREST (in the most general case) mutation rate matrix as a whole. For example, two synonymous mutations, one from A to T and one from C to T, will generally have very different rates in SARS-CoV-2 due to the fact that the underlying mutation rate from C to T is much higher than the rate from A to T. We aim to model this in our substitution rate matrix, while in the MG94 model this would not be possible. This aspect of course also differs from the GY94 model, and indeed one can think of our approach as a generalization to both the GY94 and the MG94 models. Having to choose, we think it's simpler to explain our model as an extension of the GY94 one (where we use a more general nucleotide substitution model) than as an extension of the MG94 model (where we would need to explain 2 separate differences); our choice is therefore dictated by simplicity of exposition and not by mathematical or biological principles.

Regarding the benchmarks with other softwares, it's not surprising at all that pyvolve is the slowest of the bunch (as the author of pyvolve, I'm pretty comfortable with this - it was very much not written with efficiency in mind at all...). But, I will note that pyvolve also implements Gillespie and this may affect just how slowly it runs, though it is sure to be rather slow! It would be helpful to specify whether the benchmark used Gillespie or not. I see this script in the linked github - `https://github.com/NicolaDM/phastSim/blob/main/scripts/runPyvolve.py` - which does not specify Gillespie. If one wanted to, can add argument 'algorithm = 1' when calling the evolver instance, e.g. 'my_evolver(seqfile=pathSimu+outputFile, algorithm = 1)' in version $\geq$ 1.0.0. Perhaps it could make your manuscript a bit stronger by showing, "Even with two different modes of simulating with pyvolve, it's still unreasonably slow!" :)

Answer: We thank the Reviewer for the suggestion - we were not aware of the Gillespie algorithm implementation in pyvolve. We now used the Gillespie algorithm in pyvolve, which we agree makes much more sense given the type of simulations considered here. The results presented in the new version of the manuscript (Figure 3 and S1 Text Figure S1) appear similar to those in the old version using the probability matrix approach in pyvolve.

Referee: 3

Comments to the authors

Summary. The authors develop phastSim, a software package for simulating the evolution of sequences along a tree. The authors' primary innovation is the development of a data structure that allows efficient computation of sequences on large trees. As someone who is an expert in this field, I have experimented with using a binary-search tree to efficiently identify the location of a mutation during simulation. I also abandoned such an algorithm because of the primary problem diagnosed in this paper: copying the binary-search tree to descendant phylogenetic branches is an expensive operation. The authors solved this problem by developing a multi-layer binary search tree that doesn't have to be copied at every phylogenetic split. Instead nodes maintain different views of the shared data-structure, and descendant branches add nodes to the data-structure to update their views when mutations happen without affecting other views. I found this algorithm an interesting solution to the problem.

Answer: We are thankful to the Reviewer for the comments. We address specific issues below and in the new version of the manuscript.

Major Comments. The paper's primary result is comparing features and runtime between existing programs. Such comparisons should be a secondary result in my opinion. Instead, simulation papers should focus on demonstrating the accuracy of their simulation software. However in this paper, there is no evidence presented that the simulated data generated by phastSim agrees with the models being simulated. It's not uncommon to find subtle bugs in simulation programs that introduce bias into simulations. This is why it is important for simulation papers to demonstrate their accuracy before they compare their performance to other programs. Accuracy can be demonstrated several ways, including using summary statistics, statistical tests, or parameter estimation to show that the simulated output matches what one would expect from the model. Doing all three for several different models support by phastSim would make a strong case that the software is accurate.

Answer: We previously ran some tests, some of which included in the package itself. We have now however run a more extensive series of systematic tests comparing the simulated patterns of phastSim with those of INDELible and estimating trees and substitution model parameters with RAxML from the phastSim output. The results of these tests are now included in S1 Text (Section "Testing the correctness of phastSim simulations") and confirm that the simulated patterns match expectations.

It appreciate that the code is open source and freely licensed.

Answer: We also agree that this is useful and important.

Minor Comments. Several of the algorithms presented as figures in the manuscript were adequately explained in the text. I think the paper would be improved by removing some of these algorithms from the paper. For example, Algorithms 2 and 6.

Answer: We agree that for many of the readers (for example those already familiar with search trees and with sequence simulation methods) these steps would be simple/intuitive enough not to need pseudocode. However, we also think that for many other readers it is useful to have the pseudocode explicitly representing in detail the algorithmic steps involved. Of course our opinion on the matter is not strong and we are ready to move the pseudocode in question to a supplement if deemed necessary/useful.
