# Supplementary material for: phastSim: Efficient simulation of sequence evolution for pandemic-scale datasets
Source: PLoS Comput Biol. 2022 Apr 29;18(4):e1010056. doi: 10.1371/journal.pcbi.1010056 (PMC9094560; doi:10.1371/journal.pcbi.1010056)
Supplement: Attachment — Submitted filename: responseToReviewers.pdf [file pcbi.1010056.s004.pdf]

phastSim: efficient simulation of sequence evolution forpandemic-scale datasets

Response to the Decision Letter

Please find below in blue font our response to the comments from the Reviewers.

Reviewer 1:

We thank the authors for their significant revisions. The paper looks excellent, and all of our key concerns have been addressed. We have the following (extremely minor) comments regarding the revised version of the manuscript:

Answer: We are very thankful to the Reviewer for the feedback.

- In Figures 3 and 6, it's unclear to me why "tree generation" is included in the runtime comparisons, as it's not really relevant to the task at hand (sequence simulation). I would recommend removing it such that the comparison is only between sequence simulation methods

Answer: We have now removed tree generation times from the Figures.

- In Figure 3, in the blue curve (tree generation), why is there such a large variance at the 5th point from the left? I imagine at least 1 measurement may have gotten skewed by background processes on the benchmarking machine or something; I would recommend trying to rerun that point while the machine is not being used. Note that this comment is moot if the "tree generation" curves are removed from the figures as per my previous comment

Answer: Indeed it's likely that some background process in python (memory management perhaps?) in one of the replicates might have skewed the distribution, in particular given the very short times on average of those replicates. In any case, we now removed the blue lines, as suggest in the previous comment of the Reviewer.

- In the Algorithm 6 pseudocode, at the top of the "else" statement, rather than using the syntax "int(l/2)" (which is likely the Python code that was used to typecast

the result of a floating point division to int), I would recommend using the mathematical notation for "floor", e.g. $\lfloor l/2 \rfloor$. In general, it may be good for the authors to take a pass through the algorithms to ensure that they are using standard mathematical pseudocode syntax rather than Python-like syntax where applicable

Answer: We have now fixed this.

Reviewer 2:

The authors have addressed all my comments and I am happy to recommend acceptance at this stage.

Reviewer 3:

The authors have fully addressed my comments from the previous version.

I looked at the supplemental figures showing the accuracy of the simulations. I feel that these figures were quickly put together, and the supplement would benefit from some more time spent on them. This includes (1) increasing font sizes following journal guidelines and (2) consistently marking location of no-error on all histograms. Also the histograms seem a bit blocky to me. The authors should explore other visualizations, including jitter plots, qqplots, and empirical CDFs. The authors should also make note of when two lines or plots are on top of one another, that helps readers know that data hasn't been left out.

Answer: We thank the Reviewer for the suggestions. We have now increased font sizes in the figures, and we clarify in the captions when there are overlaps in the plots. Regarding locations of no-errors, these were sometimes missing in the supplementary figures because errors are sometimes represented as distances, and so the value for error-free estimate is 0; we now include red bars also in these scenarios. We have also tried to improve the quality of the supplementary figures as suggested by the Reviewer.
